# Effects of Chaihu-Shugan-San capsule for psychogenic erectile dysfunction: Study protocol of a randomized placebo-controlled trial

**DOI:** 10.1097/MD.0000000000017925

**Published:** 2019-11-15

**Authors:** Feiqiang Ren, Ziyang Ma, Yifeng Shen, Guangsen Li, Yaodong You, Xujun Yu, Zhengjie Li, Degui Chang, Peihai Zhang

**Affiliations:** a Hospital of Chengdu University of Traditional Chinese Medicine and Chengdu University of Traditional Chinese Medicine; b The Urology and Andrology Department, Hospital of Chengdu University of Traditional Chinese Medicine; c The Andrology Department, The School of Medical and Life Sciences, Chengdu University of Traditional Chinese Medicine; d Acupuncture and Tuina School, Chengdu University of Traditional Chinese Medicine, Chengdu, Sichuan Province, PR China.

**Keywords:** Chaihu-Shugan-San capsule, protocol, psychogenic erectile dysfunction, tadalafil

## Abstract

**Background:**

Erectile dysfunction (ED) affects many adult men worldwide. Many studies on the brain of psychogenic ED have shown significant cerebral functional changes and reduced volume of gray matter and white matter microstructural alterations in widespread brain regions. Chaihu-Shugan-San (CHSGS) capsule has been used to treat ED from the 20th century in China. However, clinical research of CHSGS capsule in the treatment of ED was lack. We design this study to evaluate the efficacy and safety of CHSGS capsule in the treatment of patients suffering from psychogenic ED. Furthermore, we also aim to provide a new evidence as well as an innovation of the clinical treatment in psychogenic ED.

**Methods:**

This study is designed as a multi-center, 3-arms, randomized trial. From the perspective of psychogenic ED, we will divide patients into 3 groups, which are placebo group, tadalafil group and CHSGS group. One hundred thirty-five patients will be randomly allocated to receive placebo, CHSGS capsule or tadalafil oral pharmacotherapy. After the period of 4-week treatment, the outcome of primary assessment changes in the brain MRI, IIEF-5, EHS, and QEQ total scores from baseline. Secondary assessments include the SEAR, HAMA-14, HAMD-17 scores, response rate of the patients and their partners.

**Discussion:**

We designed this study based on previous research about psychogenic erectile dysfunction (ED). This study will provide objective evidences to evaluate the effects of CHSGS capsule as an adjuvant treatment for psychogenic ED.

**Trial registration number:**

chictr.org.cn, ChiCTR-IOR-1800018301.

## 1 Introduction

Erectile dysfunction (ED) or impotence is one of the most common male sexual dysfunctions, defined as the current or consistent presence of the disability to attain or/and maintain a penile erection sufficient for sexual satisfaction^[1,2]^ or satisfactory sexual performance which lasts for at least 6 months in accordance with the Diagnostic and Statistical Manual of Mental Disorders, Fifth Edition (DSM-5), psychogenic erectile dysfunction (pED) represents a classification of ED classification of ED due to psychological factors, is diagnosed when physical factors are excluded and serves as a significant detriment to the overall psychological well-being of patients and their partners.^[1]^

The prevalence of ED is increasing at an alarming rate in all age groups over the world, with a worldwide incidence of 12% of men under the age of 59, 22% of men between 60 and 69 years of age and 30% of men over 69 years old.^[3]^ A conservative research reported that 322 million individuals will be affected by ED around the world by the year of 2025.^[3]^ ED has been a great social problem and an important health care issue because of its huge impact upon the quality of life (QOL),^[4]^ overall psychological well-being of both sexual activity participants and its association with multiple cardiovascular diseases and atherosclerosis.^[3–8]^

According to the current guidelines, tadalafil^[9–16]^ is the first-line oral pharmacotherapy for ED. It could take action from various species in vivo and in vitro by increasing the intracellular concentrations of cGMP by amplifying the endogenous NO-cGMP pathway.^[9,17]^ The cell molecular mechanisms involved have been reviewed in detail elsewhere.^[18]^ The fact that CNS mechanisms play an important role for erection and penile as drugs targets for ED has been accepted.^[11,13,18]^ There are many studies on the brain MRI of psychogenic ED.^[7,19–31]^ Despite significant progress, the mechanism aiming at CNS targets treated by tadalafil is still unknown. Furthermore, Chinese herbs have been widely accepted for management of impotence in China for thousands of years and have been gradually recognized as a complementary therapy. According to the traditional Chinese medicine (TCM) theory, Chaihu-Shugan-San (CHSGS) could regulate the psychological factors. A systematic review showed that CHSGS can be used for the treatment for Depression.^[2]^ A case report also presented that CHSGS can relieve the anxiety symptoms. Animal studies have confirmed that CHSGS has antidepressant effect.^[32]^ Furthermore, in our clinical practice, we observed that CHSGS capsule treatment could effectively improve the symptoms and QOL of psychogenic ED patients with anxiety and depression. Psychological ED, as a typical psychosomatic disease, its occur had been proved to be related to performance anxiety, especially fear of failure during intercourse.^[1]^ Hence, we hypothesized that CHSGS could improve the symptoms of psychological ED patients and improve the quality of life by improving emotional conditions.

Normal erectile function is a result of the harmonious regulation and control of the central nervous system (CNS), which includes the supraspinal centers, the spinal cord and peripheral nerves. In the last two decades, exploring the role of CNS in sexual arousal by functional and structural neuro-imaging studies, the magnetic resonance imaging (MRI)^[20]^ such as diffusion tensor imaging (DTI),^[21,23–25]^ functional MRI (fMRI) and resting-state fMRI attracted many researchers.^[23–25]^ Since 2002, compared with healthy subjects (HS), much researchers have found that psychological ED patients showed significant changes in cerebral functions. What is more, reduced volume of gray matter and white matter microstructural alterations in widespread brain regions mediating controlled and regulated the processes of erection have also been found in many regions of the human brain of the psychogenic ED.^[21–28,33]^ MRI techniques are widely used to study the central mechanism and show positive result with premature ejaculation,^[34]^ rheumatoid arthritis,^[35]^ functional dyspepsia,^[36]^ fibromyalgia,^[37]^ addictive disorders^[38]^ and especially in depressive patients treated with paroxetine.^[39]^

Therefore, in this protocol, we plan to perform a Double-blind double-simulated randomized controlled trial, aiming to

1. compared with the placebo and tadalafil, CHSGS will be as the same effective to the treatment for psychological ED;
2. compared with tadalafil alone, CHSGS will be more effective due to its specific antidepressant effects;
3. investigate the influence of tadalafil or CHSGS capsule treatment on the brain activities of patients with psychogenic ED compared with that of placebo treatment by fMRI and
4. analyze the possible correlations between the changes of cerebral activity and the improvement of clinical variables in each group so as to explore how the tadalafil and CHSGS capsule manages depression by modulating brain function to treat psychogenic ED.

## 2 Methods/design

### 2.1 Study design

This is a multi-center, three-arms, randomized trial which was designed following the Consolidated Standards of Reporting Trials (CONSORT) Statement recommendations and the Standardized Protocol Items: Recommendations for Interventional Trials (SPIRIT) guidelines.^[40–42]^

**Figure 1:**
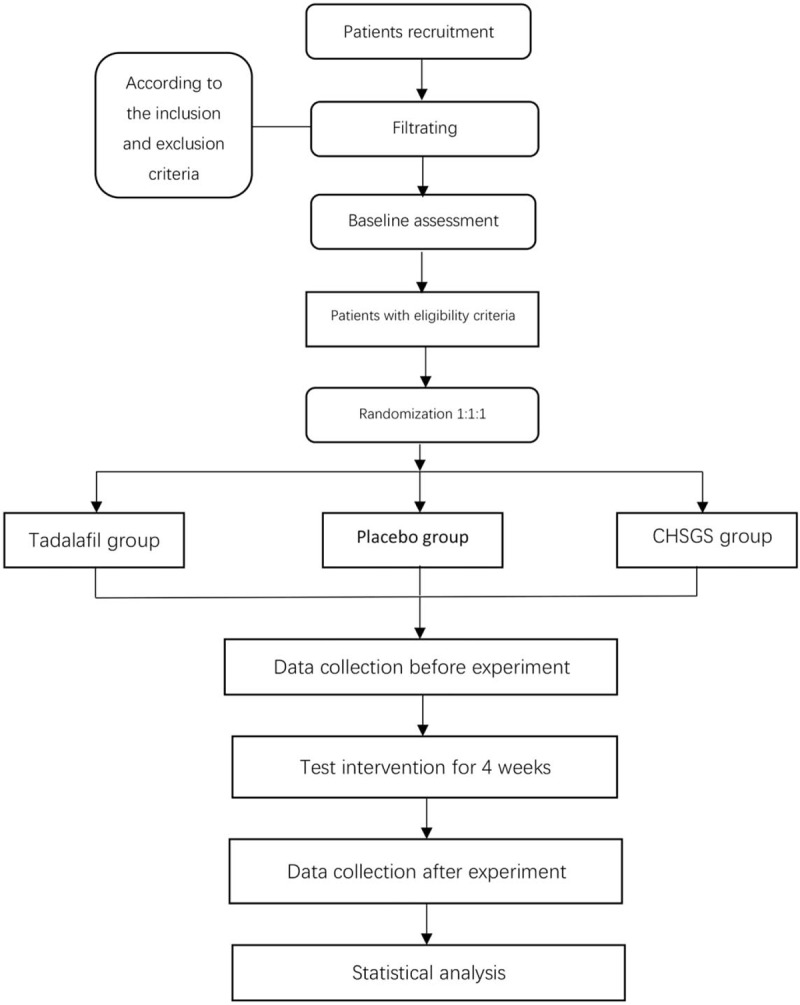
the flowchart of the study protocol.

**Figure 2:**
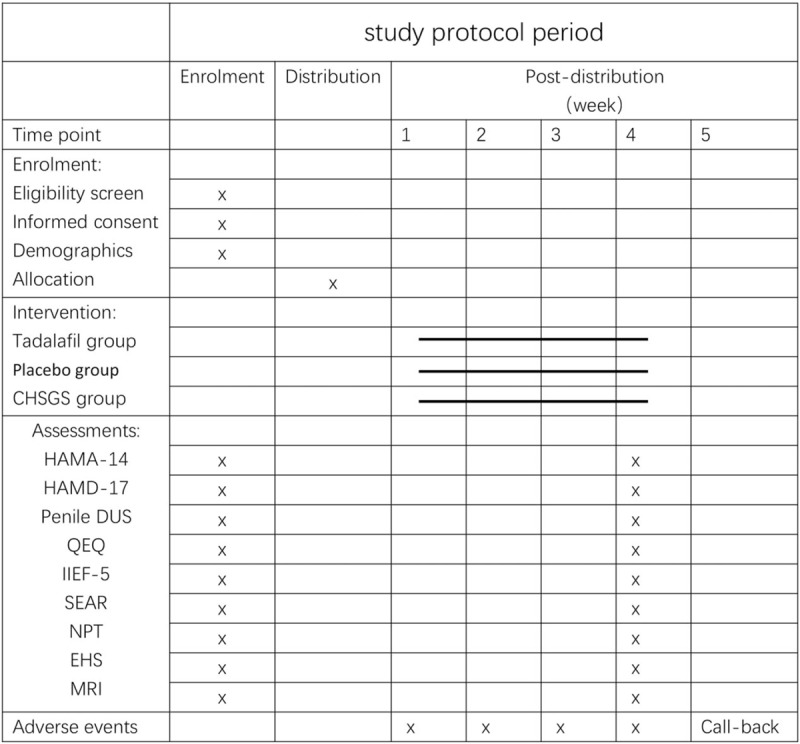
Standard protocol items. EHS = Erection hardness score, HAMA-14 = 14-item HamiJton Rating Scale for Anxiety, HAMD-17 = 17-item Hamilton Rating ScaFe for Depression, IIEF-5 = International index of erectile function 5, MRI = Magnetic resonance imaging, NPT = Nocturnal penile tumescence, penile DUS = penile Duplex doppler ultrasonography, QEQ = quality of erection questionnaire, SEAR = Self-esteem and relationship questionnaire.

This study will be carried out in the Hospital of Chengdu University of Traditional Chinese Medicine and Sichuan Integrative Medicine Hospital. During the 4-week treatment, patients in three interventions will receive a continuous oral medication with tadalafil, or CHSGS or placebo. Both the MRI scan and outcome assessment will be fulfilled at two time points (the baseline vs the end of treatment), including the baseline and the end of the intervention treatments (Figs. 1 and 2). This trial is reported in accordance with the Standard Protocol Items: Recommendations for Intervention Trials (SPIRIT) guidelines.^[40,42]^ All of the participants will be asked to sign an informed consent before the trial that will contain an introduction of pED and ED, the inclusion and exclusion criteria, and a detailed introduction to the interventions. Furthermore, participants have the right to desert the trial at any time. Ethical approval has been obtained from the Institutional Review Board (IRB) of the Hospital of Chengdu University of Traditional Chinese Medicine (Approval No. 2018KL–064) and the Sichuan Integrative Medicine Hospital (Approval No. 2017KY-02) conducted in accordance with the Helsinki Declaration, and it has been registered under the identifier No. ChiCTR-IOR-1800018301 on the Chinese Clinical Trial Registry on September 10, 2018.

### 2.2 Ethics

This study protocol has been approved by the Institutional Review Board of the Hospital of Chengdu University of TCM (Approval No. 2018KL–064), the Sichuan Integrative Medicine Hospital (Approval No. 2017KY-02) and has been registered under the identifier No. ChiCTR-IOR-1800018301 on the Chinese Clinical Trial Registry. Any modifications to the research protocol will be notified to this Human Research Ethics Committee. Informed consent will be obtained from each participant prior to enrollment. The study bases on the principles of the Declaration of Helsinki and Good Clinical Practice guidelines.

### 2.3 Recruitment

A total 135 Chinese patients who fulfill the criteria will be recruited at the Hospital of Chengdu University of Traditional Chinese Medicine (TCM) and Sichuan Integrative Medicine Hospital.

All psychogenic ED patients are recruited at the outpatient department in the Hospital of Chengdu University of Traditional Chinese Medicine (TCM) and Sichuan Integrative Medicine Hospital via advertisements in newspapers and via the Internet from November 2018 to January 2019.

All patients must be in a stable sexual relationship for at least 1 year, and must be estimated by as follows:

1. a detailed history asking, including psychosocial history (paying more attention to the patient's expectation of his own sexual performance and his general attitude and knowledge about sex, etc) and interviewing the patient's partner, medical history, mainly about relevant drug history (including tobacco, alcohol, or illicit drug use and so on), and surgical history;
2. a careful and detailed physical examination, especially the urology and andrology examination and rectify any misconceptions the patient might have about the relationship between penile length, masculinity, and erection;
3. a basic laboratory test, containing a nocturnal penile tumescence (NPT) test, a penile duplex Doppler ultrasonography (DUS) with peak systolic velocity (PSV), end-diastolic velocity (EDV) and resistant index (RI) measurements before and after intracavernous injection (ICI) of prostaglandin, as well as routine blood examinations, thyroid-stimulating hormone, serum prostatic specific antigen (PSA), and the serum sexual hormone level (sexual hormone binding globulin, free or\and total testosterone, follicle-stimulating hormone, luteinizing hormone, estrogen, and prolactin);
4. a psychophysical condition evaluated by 2 separate experienced psychologists, and
5. a series of questionnaires, mainly concerning about the International Index of Erectile Function 5 (IIEF-5), a self-esteem and relationship questionnaire (SEAR), the erection hardness score (EHS), the erection quality questionnaires (QEQ), the 14-item Hamilton Rating Scale for Anxiety (HAMA-14) and the 17-item Hamilton Rating Scale for Depression (HAMD-17). All of the above must be by checked before being enrolled in this study.

### 2.4 Sample size

Due to the lack of reference on the expected effect size of using CHSGS to treat erectile dysfunction evaluated in this study, we did not estimate the sample size based on a power calculation. According to previous studies, International Index of Erectile Function 5 (IIEF-5) scores of ED patients increased 6.0–7.1 after tadalafil.^[13,15]^ Therefore, we predicted ascension of IIEF-5 score by 6.5 points after oral tadalafil or CHSGS capsule, a reduction by 2.5 points after placebo. According to the calculation with PASS software (Version 11.0, NCSS, LLC. Kaysville, UT, USA) in a 1:1:1 ratio, when α = 0.05, 1-β = 0.8 with the standard deviation to be 3.8, the effect size is 0.2550 of 41 participants in each group. Considering a dropout rate of 10% and according to our previous study, a sample size of 135 patients at baseline will be planned in this study.

According to the sample size of previous BOLD-fMRI,^[23]^ TDI,^[21]^ and fMRI studies, 12 to 15 subjects is the very reasonable sample size with stable statistical effect.^[29,30]^ Considering the dropout rate and loss of data as to head motion during MRI scans. Among 45 patients in each group, 15 participants will be selected randomly to undergo MRI scans in this study.

### 2.5 Randomization

Qualified participants will be randomly assigned to either the tadalafil group, the placebo group or the CHSGS capsule group in a ratio of 1: 1:1. Random numbers will be generated by a random number generator in the SPSS statistical software package (Version 22.0, SAS Institute Inc), which will be operated by a third party who is uninvolved with the treatment and data collection. The drawn letters (A or B or C) will be placed into opaque envelopes labeled with sequential numbers. The envelopes will be sealed and remain in numerical order in a safe place till the completion of this study. The same researcher (not involved in the study) will prepare the envelopes.

### 2.6 Blinding

Double blind and double simulated tests will used in this study protocol to guarantee the blinding, we will made sham tadalafil and sham CHSGS capsule. The medicine will be specially handled and labeled by the Pharmacy Department in the Hospital of Chengdu University of TCM to guarantee that the patients and the practitioners included in the study will maintain completely blinded as to the identity of the treatment administered. Besides, the all practitioner will be prohibited to communicate with participants about the information of this trail.

### 2.7 Eligibility criteria

#### 2.7.1 Inclusion criteria

Patients must meet the following diagnostic criteria from the current guidelines, including:

1. men be right-handed and the age ranged from 20 to 40,
2. men be diagnosed as psychogenic ED (DSM-5),
3. men must be in a stable sexual relationship for at least 1 year with his regular sexual partner,
4. penile erection can occur in the circumstance of masturbation or audiovisual stimulation,
5. men who meet the TCM diagnosisof stagnation of liver-qi”,
6. men who agree to complete all demand during the study period,
7. sign an informed consent form.

#### 2.7.2 Exclusion criteria

Exclusion criteria for the forthcoming study are as follows:

1. be diagnosed as organic ED rather than psychogenic disorder,
2. a current diagnosis or history of drug or alcohol dependence;
3. use of any medication that might impact sexual function during the previous 30 days before enrolled in this study;
4. any physical illness as assessed by personal history and laboratory analysis finding any history of serious psychiatric, neurological, cardiovascular, respiratory, gastrointestinal or renal diseases or hepatic disorders or significant physical disorders;
5. participated in any other current clinical trials;
6. had any contraindications for MRI scan.

### 2.8 Test drugs

Test drugs are tadalafil, CHSGS capsule and placebo, provided by the Hospital of Chengdu University of TCM. The three drugs had the same shape, size, color, smell, package, and Lot number.

## 3 Intervention

### 3.1 CHSGS group

Patients will be provided three-daily treatment with 5 g CHSGS added to the 5 capsule each time until 4 weeks.

The CHSGS preparation contained the gathering of the following components: 6 g Bupleurum Chinese root, 4.5 g Rhizoma Chuanxiong, 4.5 g Fructus Aurantii, 6 g Pericarpium CitriReticulatae, 4.5 g Paeonia, 1.5 g Glycyrrhizaeuralensis root and 5 g Cyperusrotundus. All CHSGS herbal components were obtained from the Hospital of Chengdu University of TCM. The herbal components were identified by an expert in order to fulfill the quality requirements of the Pharmacopoeia of the People's Republic of China (2015 edition). CHSGS and its components were individually decocted in boiling water for 30 minutes, concentrated and vacuum-dried to form a paste, and were subsequently combined into a paste containing 8 g crude extracts per gram. Starch powder was mixed into a container. Stir it well and make it into capsule.

### 3.2 Tadalafil group

Patients will be provided a once-daily treatment with 5 mg tadalafil added to the capsule until 4 weeks.

According to the current guidelines, the therapeutic interventions for ED are basic treatment, drug treatment, physical treatment and surgical treatment.^[12]^ Drugs are phosphodiesterase type 5 inhibitors such as tadalafil, sildenafil, vardenafil, avanafil, which have been regarded as the first-line oral pharmacotherapy for ED.^[17,18]^ Some previous clinical research have confirmed that patients and partners prefer tadalafil to sildenafil in the treatment of ED.^[11,13–16,43,44]^ Therefore, we selected tadalafil as the positive control group as well as the treatment group. Smashed tadalafil will be mixed with starch powder and stir well into a container in order to be made into capsule.

### 3.3 Placebo group

Patients will be provided three-daily treatment with the 5 g mixed starch powder added to the 5 capsule each time until 4 weeks.

It is known that placebos are the best comparison between clinical controlled trials. Therefore, we adopted this placebo and standardized its performance. The placebo components were obtained from the Hospital of Chengdu University of TCM. The standard operating procedures (SOP) are listed as follows:

1. the materials are starch,
2. add the liquid base: place the mixed starch powder into a container, add honey and stir well to make it into capsule. The above materials made shared the same character with the CHSGS capsule in terms of appearance, weight and taste.

### 3.4 MRI scan

The MRI scanning of the brain will be executed on a 3.0-T GE MRI scanner (General Electric Company, America) at the Hospital of Chengdu University of Traditional Chinese Medicine, Chengdu, China, including the magnetic resonance (MR) diffusion tensor imaging (DTI), T1-weighted, a resting-state blood oxygenation level dependent MRI (BOLD-MRI) data of all participants in this study.

T1-weighted structure images in 3D Magnetization-Prepared Rapid-Gradient-Echo (MPRAGE) Images will be firstly collected before resting-state scanning with the following sequence parameters: field of view (FOV) = 240 × 240 mm^2^, repetition time (TR) = 1900 ms, echo time (TE) = 2.26ms, and matrix = 256 × 256.

DTI will be applied along 63 non-linear directions (*b* value = 1000 s/mm2) together with an acquisition without diffusion weighting (*b* value = 0 s/mm^2^), and the scanning parameters are as follows: FOV = 230 × 230 mm^2^, TR = 8900 ms, TE = 84 ms, and matrix = 256 × 256.

The resting-state BOLD-MRI will be obtained by using Gradient-Recalled Echo-Planer Imaging (GER-EPI) with the following sequence parameters: TR = 2000 ms, TE = 30 ms, inverse angler (IA) = 90°, number of slice = 32, slice thickness (ST) = 5 mm, FOV = 240 × 240 mm^2^, matrix = 64 × 64, and total volumes = 400.

### 3.5 Outcome assessment

The following outcomes will be assessed by independent assessors, who had been trained before participating in this study and blinded to the randomization. All outcome data for participants whether completed or withdrawn during the study will be collected and recorded in the case report form (CRF).

#### 3.5.1 Primary outcome

Based on various clinical studies, all the patients, including the primary symptoms of the ED patients will be assessed on mean changes from baseline (the day that the patient begin protocol) to end of observation (the day of end of protocol) in the brain MRI and International Index of Erectile Function 5 (IIEF-5) total scores,^[45,46]^ which has been widely used and recommended as a primary outcome for clinical trials of ED. Furthermore, the quality of erection questionnaire (QEQ)^[47–49]^ and the erection hardness score (EHS) total scores will also be included in this study.^[47,50]^

#### 3.5.2 Secondary outcome

In terms of the assessment of symptom severity of erectile function for all the participants in this study, the secondary outcome will be based on the self-esteem and relationship (SEAR) questionnaire.^[51]^ As to the level of anxiety and depression of all the participants in this study, it will be evaluated by two independent psychologists within the questionnaires of the 14-item Hamilton Anxiety Rating Scale (HAMA-14) and the 17-item Hamilton Depression Rating Scale (HAMD-17).^[52,53]^

### 3.6 Adverse events

Adverse events (AEs) related to tadalafil treatment such as myalgia, back pain and flushing will be appropriately estimated and recorded by the observers during the trial. AEs will be managed by a specialized practitioner (do not participate in clinical data analysis) within 24 hours. The principal researcher (PH Zhang) will make the final decision to terminate the trial if severe AEs occurs. All data for participants during the study and details of related and unexpected AEs (the time of occurrence, severity of AE, and suspected causes) will be collected and recorded in the case report form (CRF). Measures may vary from symptomatic treatment to case submission to the Research Ethics Committee within 48 hours depending on the severity of AEs.

### 3.7 Quality control and Data collection

Due to the fact that any nonstandard or bias input of clinical data can dominate the bias of results, two practitioners (to estimate the effect of treatment and the data authenticity) will independently gather the data with case report forms and original cerebrum MRI. The collected data will be input into a dedicated computer. The above process will maximize the reliability and safety of the all data. In order to guarantee the quality of the study, all practitioners will be required to have an official license for at least 2 years of protocol study and clinical experience.

### 3.8 Data analysis

#### 3.8.1 Clinical variables analysis

The clinical data will be analyzed with the help of SPSS22.0 (SPSS Inc., Chicago, IL) by 2 blinded evaluators. The missing data analysis will be based on the intention-to-treat (ITT) principle regarding baseline characteristics. The clinical scores of the subjects (including the pED severity and duration, the sum scores of IIEF-5, SEAR, EHS, QEQ, HAMD-17 and HAMA-14) will be examined by means of the Pearson correlation analysis. All the clinical data in this study will be presented as follows:

1. a continuous measurement data being presented as the mean±standard deviation (SD), the mean, SD, median and interquartile range.
2. a categorical data being presented as case and percentages. Analysis tools include: the Cochran-Mantel-Haenszel (CMH) test or nonparametric test, an independent samples t-test, and a two-sided test. Also, analysis of covariance, covariance analysis or generalized estimating equations, *χ*^2^ and Linear regression will be applied on all available and suitable data (*P* value <.05 is considered to be statistically significant).

#### 3.8.2 MRI data analysis

The fMRI scan data will be preprocessed and analyzed by SPM12 software (http://www.fil.ion.ucl.ac.uk/spm/) performed on MATLAB 2015b (MathWorks, Inc, Natick, MA). Two-sample *t* tests will be used to evaluate possible cerebral responses in each group by within-group analysis (post-treatment minus pre-treatment). We will use between-group analysis to compare the difference in cerebral response changes. Correlation analysis will be conducted to investigate changes between fMRI image data and corresponding clinical data in each group. Based on our previous research's methods and acquisition of the brain MRI data, MRI data will be firstly preprocessed and analyzed by SPM12 software performing on MATLAB 8.6 (Math works, Inc., Natick, MA, USA), Brain Voyager QXsoftware (Brain Innovation, The Netherlands).^[54]^ And then, for accurately investigate the different cerebral responses, including regional homogeneity (ReHo),^[55]^ tract-based spatial statistics (TBSS),^[56,57]^ amplitude of low-frequency fluctuation (ALFF),^[58]^ and functional connectivity (FC) will be used in this study. Finally, correlation analysis will be processed between clinical data and MRI image data.

**Figure 3:**
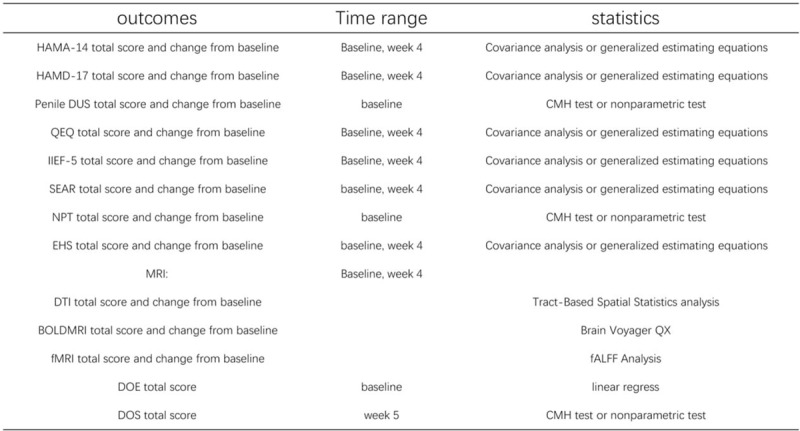
Analysis methods for outcomes. BOLDMRl = Bipod oxygenation level dependent MRI, CMH = Cochran-Mantel-Haenszel test, DOE = Degree of expectation, DOS. Degree of satisfaction, DTI = Diffusion tensor imaging, EHS = Erection hardness score, fALFF = Fractional Amplitude of Low-Frequency Fluctuation Analyses, fMRI = functional MRI, HAM A-14 = 14-item Hamilton Rating Scale for Anxiety, HAMD-17 = 17-item Hamilton Rating Scale for Depression, IIEF-S = International index of erectile function 5, MRI = Magnetic resonance imaging, NPT = Nocturnal peniEe tumescence, penile DUS = penile Duplex doppler ultrasonography, QEQ = Quality of erection tjuesironnaire, SEAR = Self-esteem and relationship questionnaire.

Figure 3 illustrates the data access and the analysis of the brain MRI data as well as the analysis methods of the primary and secondary outcomes.

## 4 Discussion

In this protocol study, the qualified participants are asked to maintain their current living habits and sexual performances, especially their diet, exercise and the quality of sleep. Meanwhile, they are required to fill out relevant questionnaires throughout the full observational process. To satisfy the requirements of the Rule of Ethics and reduce the bias caused by performance, the rest of the all clinical procedures maintain the same.

Seen from the current records and recent RCT results, tadalafil has an enduring effect and was the best-marched choice between patients and their partner for treating psychogenic ED. However, its central mechanism remains unclear. Considering the importance of the brain for the psychogenic ED, we have designed the randomized controlled MRI trial to reveal the central mechanism of the psychogenic ED treated by tadalafil. In this study, we will compare the changes in brain function after administration of 2 interventions: the tadalafil and the CHSGS capsule.

**Figure 4:**
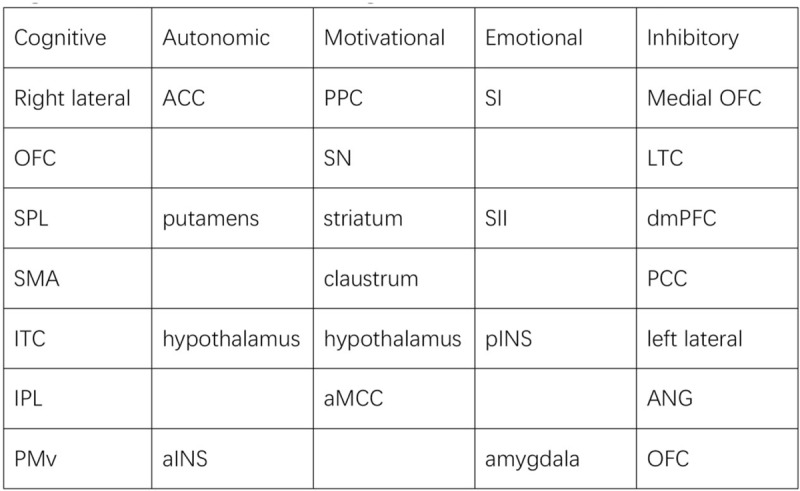
The central transmitters in brain regions. ACC = anterior cingulate cortex, aINS/plNS = anterior/posterior insula, aMCC = anterior middle cingulate cortex, ANG = angular gyri, dmpFC = dorsal medial prefrontal Cortex, IPL/SPL = inferior/superior parietal lobule, ITC = inferior temporal cortex, LTC = lateral temporal cortex, OFC = orbitofrontal cortex, PCC = posterior cingulate cortex, PMv = ventral premotor area, PPC = posterior parietal cortex (Brodmann area 40), SI/SII = primary/secondary somatosensory cortex, SN = substantia nigra, SWA = supplementary motor area.

Many regions of the human brain have been found to play a very important role in human sexual arousal as well as penile erection, emotion, and cognition. Over the last 15 years, MRI science has been widely used to disclose the cerebral mechanism of human sexual arousal especially the penile erection and investigate the brain function alteration in psychogenic ED and healthy subjects. Erection is known to be one of the most convincing and mischievous signs of the sexual response cycle. Relatively, ED not only can be conceptualized as an impairment in the arousal phase of sexual response,^[59]^ but also can be seen as a central and spinal center reflex, which can be sponsored by recruitment of penile afferents, both somatic and autonomic, and the changes of visual, imaginary stimuli, and olfactory. Several central transmitters^[60]^ play a crucial role in erectile control and the main regions that control of male sexual arousal are summarized in Figure 4. Previous DTI studies showed that psychogenic ED had decreased or increased white matter microstructures in multiple brain regions.^[21]^ Besides, brain networks neuro-imaging studies also had showed that psychogenic ED had increased the small-worldness and modules in the left prefrontal cortex and limbic system including the right superior parietal gyrus, and the superior parietal gyrus.^[23–25]^These brain networks were absolutely believed to be closely related to the brain sexual arousal of the psychogenic ED.^[60–63]^

In order to guarantee the reliability of the result of this study, we adopt the quality key point control as follows:

1. patient in this study restricts the subjects to age between 20 to 40 and being right-handed for baseline homogeneity;
2. sample size to obtain stable statistical power; 135 patients will be included in each group for clinical evaluation and 15 patients will be included in each group for the central mechanism study and receive MRI scans; and
3. MRI scans; each scan will be performed in the afternoon with the same scanner and operator. During the scanning period, all participants will be asked to remain relaxed, keep their eyes closed and wear a birdcage head coil filled with sponge material and to stay still to reduce the effects of head movement. The scanning room maintains the noise less than 150 dB and the temperature between 18 and 22°C, with humidity higher than 60%.

In conclusion, this study is conducted in the purpose of supporting the concept of brain MRI alteration in psychogenic ED with tadalafil or CHSGS capsule. We expect that the results of this trial can provide both an evidence-based treatment option for patients suffering from ED, an enhanced level of evidence on which central mechanism research and to provide an innovation of the clinical treatment in psychogenic ED.

## Author contributions

FR, ZM, PZ, DC, YS and ZL have put forward the conception and design of the Trial and planned for the analysis of the data. FR, PZ, YS and DC agreed in drafting this manuscript. FR, ZM, XY, YY, DC, and GL, standing in the data Collection, ZL standing in the data analysis, and were in the charge of recruitment and treatment of patients. Authors discussed, read, revised the manuscript, and approved the final manuscript.
